# Fidelity of implementation of TB screening guidelines by health providers at selected HIV clinics in Ghana

**DOI:** 10.1371/journal.pone.0257486

**Published:** 2021-09-17

**Authors:** Solomon A. Narh-Bana, Mary Kawonga, Esnat D. Chirwa, Latifat Ibisomi, Frank Bonsu, Tobias F. Chirwa

**Affiliations:** 1 School of Public Health, Faculty of Health Sciences, University of the Witwatersrand, Johannesburg, South Africa; 2 Dodowa Health Research Centre, Research and Development Division, Ghana Health Service, Accra, Ghana; 3 Department of Community Health, Charlotte Maxeke Johannesburg Academic Hospital, Johannesburg, South Africa; 4 Gender & Health Research Unit, Medical Research Council, Johannesburg, South Africa; 5 Nigerian Institute of Medical Research, Yaba, Lagos, Nigeria; 6 National TB Control Programme, Accra, Ghana; University of Washington, UNITED STATES

## Abstract

**Introduction:**

Tuberculosis screening of people living with human immunodeficiency virus is an intervention recommended by the WHO to control the dual epidemic of TB and HIV. The extent to which the intervention is adhered to by the HIV healthcare providers (fidelity) determines the intervention’s effectiveness as measured by patient outcomes, but literature on fidelity is scarce. This study assessed provider implementation fidelity to national guidelines on TB screening at HIV clinics in Ghana.

**Methods:**

It was a cross-sectional study that used structured questionnaires to gather data, involving 226 of 243 HIV healthcare providers in 27 HIV clinics across Ghana. The overall fidelity score comprised sixteen items with a maximum score of 48 grouped into three components of the screening intervention (TB diagnosis, TB awareness and TB symptoms questionnaire). Simple summation of item scores was done to determine fidelity score per provider. In this paper, we define the level of fidelity as low if the scores were below the median score and were otherwise categorized as high. Background factors potentially associated with implementation fidelity level were assessed using cluster-based logistic regression. Odds ratio with 95% confidence interval (CI) was used as the measure of association.

**Results:**

Of the 226 healthcare providers interviewed, 60% (135) were females with a mean age of 34.5 years (SD = 8.3). Most of them were clinicians [63% (142)] and had post-secondary non-tertiary education [62% (141)]. Overall, 53% (119) of the healthcare providers were categorized to have implemented the intervention with high fidelity. Also, 56% (126), 53% (120), and 59% (134) of the providers implemented the TB diagnosis, TB awareness and TB symptoms questionnaire components respectively with high fidelity. After adjusting for cluster effect, female providers (AOR = 2.36, 95%CI: 1.09–5.10, p = <0.029), those with tertiary education (AOR = 4.31, 95%CI: 2.12–9.10, p = 0.040), and clinicians (AOR = 1.78, 95%CI: 1.07–3.50, p = 0.045) were more likely to adhere to the guidelines compared to their counterparts.

**Conclusion:**

The number of providers with fidelity scores above the median was marginally greater (6%) than the number with fidelity score below the median. Similarly, for each of the components, the number of providers with fidelity scores higher than the median was marginally higher. This could explain the existing fluctuations in the intervention outcomes in Ghana. We found gender, profession and education were associated with provider implementation fidelity. To improve fidelity level among HIV healthcare providers, and realize the aims of the TB screening intervention among PLHIV in Ghana, further training on implementing all components of the intervention is critical.

## Introduction

Globally, tuberculosis (TB) disease is known to be the main cause of death among people living with HIV (PLHIV) [1]. PLHIV are more likely to succumb to TB diseases than people without HIV due to their compromised immune system. Intensified TB case-finding through TB screening of PLHIV is one of the World Health Organization (WHO) policies emphasizing TB and HIV collaborative activities as a means to reducing the dual burden of TB and HIV [2, 3]. Effective TB screening of PLHIV ensures early identification of those with presumptive TB infection who requires a confirmatory test through X-ray or genXpert for appropriate treatment [1]. The early detection helps to identify TB cases and ensures the separation of cases from non-cases to reduce the TB transmission in PLHIV [1].

The National TB Control Programme (NTP) in Ghana, like many other countries where HIV and TB are of public health concern, adopted and implemented the policy on TB screening of PLHIV in 2004 [2, 4]. PLHIV who attend HIV clinics should be systematically and routinely screened for TB at every visit using the WHO-approved screening questionnaire for the presence of cough of any duration, fever, weight loss and night sweats [5]. PLHIV with the presence of one or more of these symptoms have a confirmatory test (X-ray/XpertMTB/RIF) and then appropriate preventive or curative treatment depending on the result [3]. Although the intervention (TB screening of PLHIV) was implemented over a decade ago [6] the proportion of PLHIV screened had not been stable–declines and fluctuates over time [7]. In the early years of the implementation, the proportion of PLHIV screened for TB was about 70–96% [7]. But this fluctuated as the years progressed, with about 80% of PLHIV in 2013 [7] and 41% in 2014 [8] not screened for TB. This led to a review of the policy in 2014 with new screening targets set at 56% for 2015, and progressively increasing the target to 80% in 2018 and 90% in 2020 [7]. In 2018, 44000 TB cases and 15,800 TB deaths were recorded with an estimated incidence rate of 148 per 100,000 population [9]. Also, according to the WHO global TB report (2020) [10] out of 35,424 newly enrolled person in HIV care in Ghana, 2620 (7.4%) were notified as TB cases.

Intervention implementation under real-life conditions is known to be complex and influenced by different factors [11–13]. The implementation science (IS) literature posits that interventions of proven effectiveness may fail to yield intended outcomes due to challenges related to the implementation of the intervention in real-world settings [14–17].

Some researchers postulate that interventions yield the desired outcome if the implementation is effective [17–19]. One of the ways to measure implementation effectiveness is to assess what is referred to in IS as implementation fidelity (IF) (that is the degree to which the intervention is being implemented by healthcare providers as intended by the designers of the intervention) [20–24]. Some researchers have conceptualized how different factors act or interplay to influence the implementation programme [12, 14–16, 25, 26]. Carroll et al. 2007 [24] developed a comprehensive framework outlining the components of implementation fidelity as adherence covering activities of content, coverage, frequency and duration. It also outlined that the level of implementation fidelity may be influenced by factors called moderators subcategorized as intervention complexity, facilitation strategies quality of delivery, and participant’s responsiveness. They emphasized that IF is the same as adherence and so therefore, the assessment of IF is tantamount to adherence assessment. It is argued that any of these fidelity components alone, or a mix depending on the intervention and the guideline, could be used to measure implementation fidelity [21, 27]. But the mix must show consistency in dimensionality (number of attributes of a dataset for an outcome), reliability (produce the same finding under similar situation) and validity (measures what it is intended to) through the use of an empirical method of analysis such as structural equation modelling, factor analysis and principal component analysis [28, 29], summation of items/activities/component scores [30]. These analytic techniques are helpful to researchers in determining the best combination of items. Interventions are aided with policy or programme guidelines based on both theoretical and empirical grounds. These guidelines contain the core components of the intervention without which the intervention cannot operate effectively to achieve the intended outcome [31, 32].

Many studies have been conducted on IF from different disciplines [33]; in education [34], social work [35], engineering [36], construction [37] and health [16, 38, 39], the area of this study. IF is important in the process of translating evidence-based interventions into real-life practice. This means, whether an intervention is implemented within the health sector according to design, for instance, has repercussions on whether that intervention will achieve in the population the health outcomes observed in the clinical trials [15, 19]. However, there is a dearth of research on the fidelity of implementation of the TB screening intervention among PLHIV in Ghana or elsewhere. Most studies conducted in this area are focused on screening coverage [40–42]. Another one was a before-and-after study that assessed the impact of integrating TB and HIV services on TB treatment outcomes and explored the usefulness of TB treatment outcomes as TB/HIV indicators [43]. Whether and the extent to which the TB screening intervention among PLHIV is being implemented would have an impact on the coverage of this intervention among PLHIV (service outcome) and ultimately the burden of TB among PLHIV (health outcome). However, no study has been conducted in Ghana to assess IF as a possible reason for the observed low and fluctuating screening coverage outcomes since the implementation of the intervention. This study, therefore, assessed the level of provider IF to TB screening among PLHIV attending HIV clinics in Ghana as well as factors associated with IF level.

### Conceptual framework: The conceptual framework for implementation fidelity

Many frameworks have been developed for assessing IF. Where applicable, some studies combine adherence and competence to assess IF [38, 44, 45] while **other** studies assessed only adherence and consider competence as a moderating factor [46, 47]. Having identified adherence as our focus of assessing IF for this study, we adapted Carroll *et al*. Conceptual Framework for Implementation Fidelity (CFIF) [24] because it harnessed other frameworks and is a comprehensive and useful way of assessing IF [12]. Carroll et al. [24] reviewed existing theories and frameworks of IF and developed the CFIF with constructs guiding the assessment and understanding of implementation fidelity. The framework was made up of five elements; adherence, dose, quality of delivery, participant responsiveness, and programme differentiation. The framework suggested that the measurement of adherence (content, frequency, duration and coverage) to the intervention is IF. It considers the rest of the elements as moderating factors of fidelity level achieved. The desired outcome of TB screening depends largely on how well the intervention is implemented. The final implementers of the intervention were the providers at the HIV clinics.

We, therefore, adapted and drew conceptual inspiration from CFIF to assess IF for this study at the provider level. According to Carroll et al. [24], successful implementation can only result if the essential components of the intervention are identified and implemented as planned. The identification offers the opportunity for adaptation. Clinical guidelines outlining what the healthcare providers are expected to do in terms of TB screening among PLHIV exist [2, 3, 7]. In adapting the CFIF to measure IF, we reviewed the TB screening guideline among PLHIV and identified essential components. These components were grouped under content and frequency sub-constructs of adherence.

**Fig 1:**
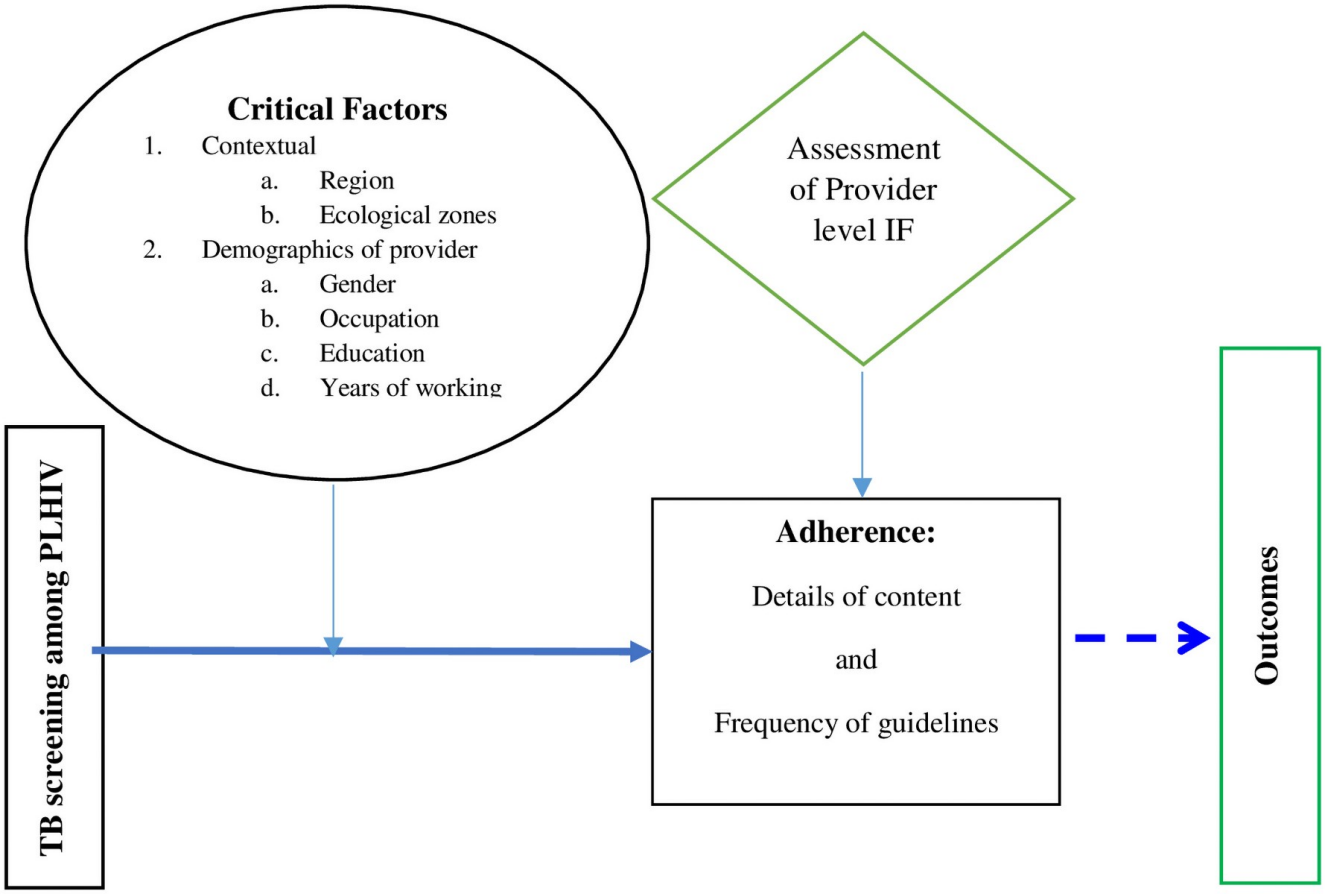
A conceptual framework for provider level IF to TB screening at HIV clinics in Ghana: Adapted from CFIF by Carroll et al., 2007.

The level of IF is influenced by the relationship between other factors and the essential components of the intervention identified. In our study, the IF could potentially be influenced by an interaction between those essential components and critical characteristics such as contextual factors (regional location of the HIV clinic) and the provider characteristics. They are widely considered critical because they influence attitude and motivation which eventually affects behaviour. Based on the concept of the framework, we finally decided to examine the influence of these critical characteristics on IF as depicted in Fig 1. The broken line in Fig 1 shows that the connection between TB screening among PLHIV and its outcomes is external to IF, but that the degree of IF attained can alter this relationship.

The details of content and frequency sub-constructs used in this study are defined as below:

1. Content–the degree to which healthcare providers at the HIV clinic follow the intervention guideline, e.g. providing important counselling and education, screening, referral, and appropriate treatment
2. Frequency–the extent to which healthcare providers at the HIV clinic perform the activities on the guideline according to the prescribed frequency, e.g. how often does the HIV care provider conducts counselling, education, use the TB screening tools among PLHIV; which must be conducted all the time for all clients seen.

## Materials and methods

### Ethics statement

Protocol approvals were obtained from both the Human Research Ethics Committee (Medical) of the University of the Witwatersrand, Johannesburg, South Africa (Clearance number M190110) and the Ghana Health Service Ethical Review Committee (GHS-ERC), Accra, Ghana (clearance number GHS-ERC002/01/19). All participants in the study gave written consent.

### Study site

Ghana administratively is made up of 10 regions subdivided into 216 districts in 2017. Out of the 216 districts, 140 have district hospitals. This study was conducted in 27 HIV clinics located within 27 district hospitals across the 10 regions. These 27 district hospitals have and mandatorily run HIV clinics daily on all the 5-working days of the week. These district hospitals were chosen for this study because as part of care, the NTP identified these 27 district hospitals with both X-ray machines and geneXperts for TB confirmatory tests as the first sites to begin the implementation of isoniazid preventive therapy (IPT) for screened PLHIV who tested negative for TB.

**Fig 2:**
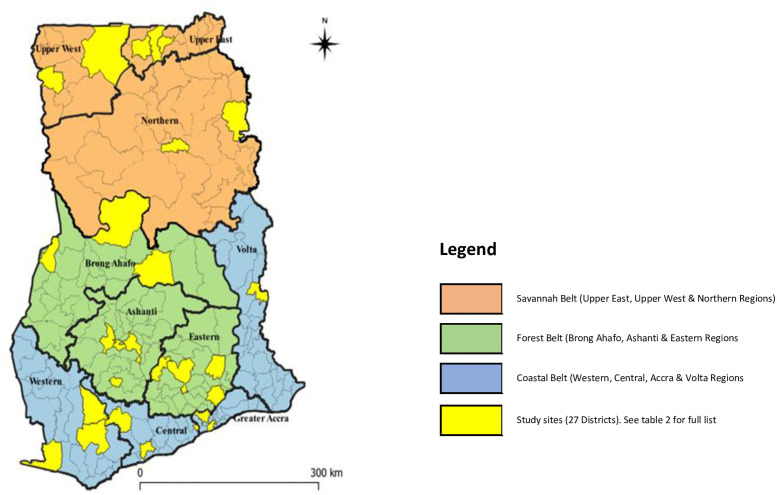
A map of Ghana showing the 10 regions and the study sites.

The regional distribution of these hospitals are Volta 1; Upper West, Northern, and Greater Accra with 2 each; Brong Ahafo, Central, Upper East and Western with 3 each and Eastern and Ashanti with 4 each as shown on the map of Ghana (Fig 2).

Among PLHIV, preventing TB involves the three ‘I’s: Intensified case finding, Isoniazid Preventive Therapy (IPT), and Infection Control for TB including early initiation of ART. At the HIV clinic within the district hospital, trained healthcare providers (clinical and non-clinical) guided by the guidelines for TB preventive therapy in Ghana provide TB preventive services to PLHIV attending the clinic. At every visit, PLHIV are given TB counselling and screening using TB symptom screening questionnaire. Based on the client response to the signs and symptoms (cough of any duration, night sweat, chest pain, weight loss or fever) providers had to refer client to for GenXpert or X-rays confirmation test. If *Mycobacterium* TB is detected, providers initiate anti therapy appropriately, otherwise they initiate TB preventive therapy based on TB expert evaluation.

**Fig 3:**
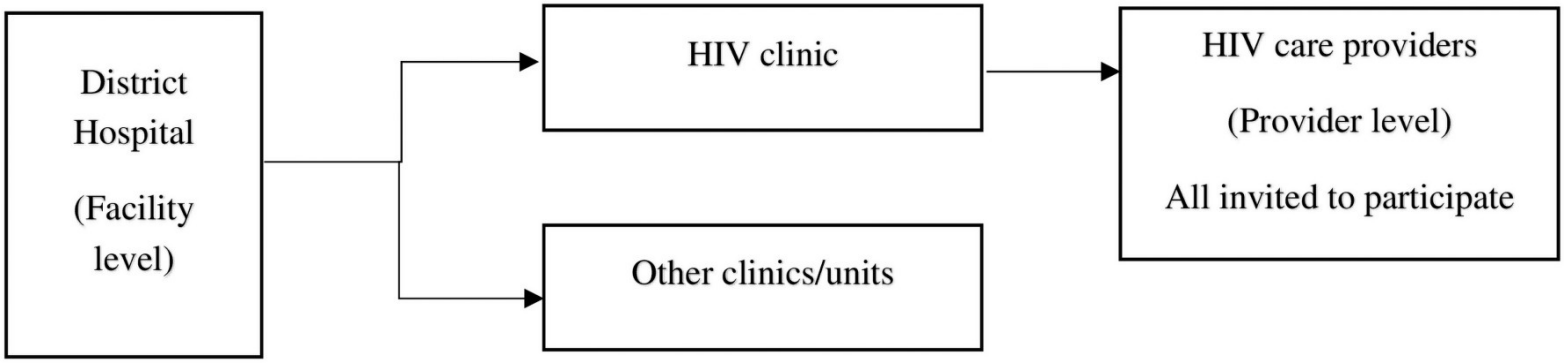
A typical structure of district hospital depicting where facility and provider levels belonged.

Unlike other sectors like education, construction, and engineering, the health sector at the district hospital level (Fig 3) for instance had intervention guidelines for *(1)* management at facility or programme level and *(2)* providers who are the direct implementers of the intervention to participants. The structure in Fig 3 refers to the arrangement or formation at the hospital. All the providers at the HIV care clinics were equipped to screen PLHIV for TB. The NPT ensured that all HIV clinics staff had engaged in either a pre-service, in-service, and or continuing professional development and specialization course training on TB and HIV issues including TB screening. These providers include clinicians (provide direct care to patients, e.g. nurses and physicians) and non-clinicians (support patient care, e.g. disease control, task-shifting, health promotion, and nutrition officers). Therefore both the clinicians and non-clinicians at the HIV clinic trained on TB collaborative activities are required to provide TB and HIV counseling, conduct TB screening with the TB screening questionnaire, administer ARV, and provide other TB related activities among PLHIV at HIV clinics. Non-clinicians exclude laboratory and pharmacy staff who might have received trainings on TB related activities but do not do TB screening.

### Study design and sampling

A total of 27 HIV clinics within the identified 27 district hospitals with both the X-ray machines and geneXperts for TB confirmatory tests were surveyed for the study.

The study was cross-sectional that used a census-based survey to interview all the providers at the 27 HIV care clinics. This census-based method was applied to achieve a good number of participants to conduct a meaningful analysis of the study. To maximize representation and ensure all providers involved in doing TB screening at the clinic participated in the study, the number of providers per clinic was established with an effort to include everyone in the study. Staff and other healthcare providers who did not work directly with the HIV clinic were excluded.

### Data collection

A questionnaire was developed and used to collect data from the participants. The structured questionnaire collected data on the demographic characteristics of the provider, and data on their reported adherence to the clinical guidelines on screening of PLHIV, focusing on contents and frequency of the intervention implementation. Prior information and knowledge from the TB/HIV collaborative protocol guideline by the Ghana NTP [6] together with the CFIF guided the measurement approach. The provider-level fidelity questionnaire was developed based on the key elements or activities in the guidelines for the provision of TB screening among people living with HIV attending the HIV care clinic. The questionnaire focused on the main activities directly relating to TB screening by the providers, as documented in the guideline. A total of 19 questions were formulated from these main activities and as mentioned earlier was part of the pretest during the research assistants training.

As with many other healthcare surveys [48, 49], most of the questions were formulated on different point-Likert scales depending on what it sought to measure [50]. Table 1 presents a list of questions as outlined in the TB screening guideline and grouped under each of the content and frequency sub-elements and how they were measured.

**Table 1:** List of the 19 items used to determine provider implementation fidelity score.

Content items			Frequency items	
Description *(variable name)*		Mean	Description *(variable name)*	Mean
***Response*:**			***Response*:**	
			1 = Never	
			2 = Rarely	
No…..0	Yes….1			
			3 = For some clients sometimes	
			4 = Either for all new clients or annually for existing clients	
			5 = All the time for all clients	
**Component 1: TB diagnosis**				
Asks client about cough of any duration.		0.863	How frequently do ask PLHIV about cough of any duration during consultation?	4.257
Asks if the client has night sweats.		0.686	How frequently do you ask PLHIV about night sweats during consultation?	3.580
Asks if the client had fever.		0.619	How frequently do you ask PLHIV if they had a fever during consultation?	3.553
Asks if the client has pain in the chest.		0.748	How frequently do you ask PLHIV about pain in the chest during consultation?	3.823
Asks/checks if the client lost weight.		0.673	How frequently do you ask/check the weight of PLHIV during consultation?	3.336
**Component 2: TB awareness**				
Provides education.		0.659	How frequently do you provide education for PLHIV?	3.385
Provides counselling about TB screening.		0.726	How frequently do you provide counselling about TB screening for PLHIV?	3.695
**Component 3: TB symptoms questionnaire**				
Uses TB screening questionnaire (clinical algorithm).		0.730	How frequently do you use the TB screening questionnaire for PLHIV?	3.668
**Component 4: TB screening process**				
Do you ask all the HIV clients all the TB screening questions?		0.664		
After screening the client for TB, what do you do next? ^^^				2.0
1 = No adherent 2 = Partially adherent 3 = fully adherent				
If you identify a client who you presumed of having TB, what steps would you take? ^^^				1.650
1 = Not adherent 2 = Partially adherent 3 = fully adherent				

Note:

^^^ Open-ended questions.

The means of each items were computed based on the responses from the providers to give prior information on each items prior to further analysis.

**Fig 4:**
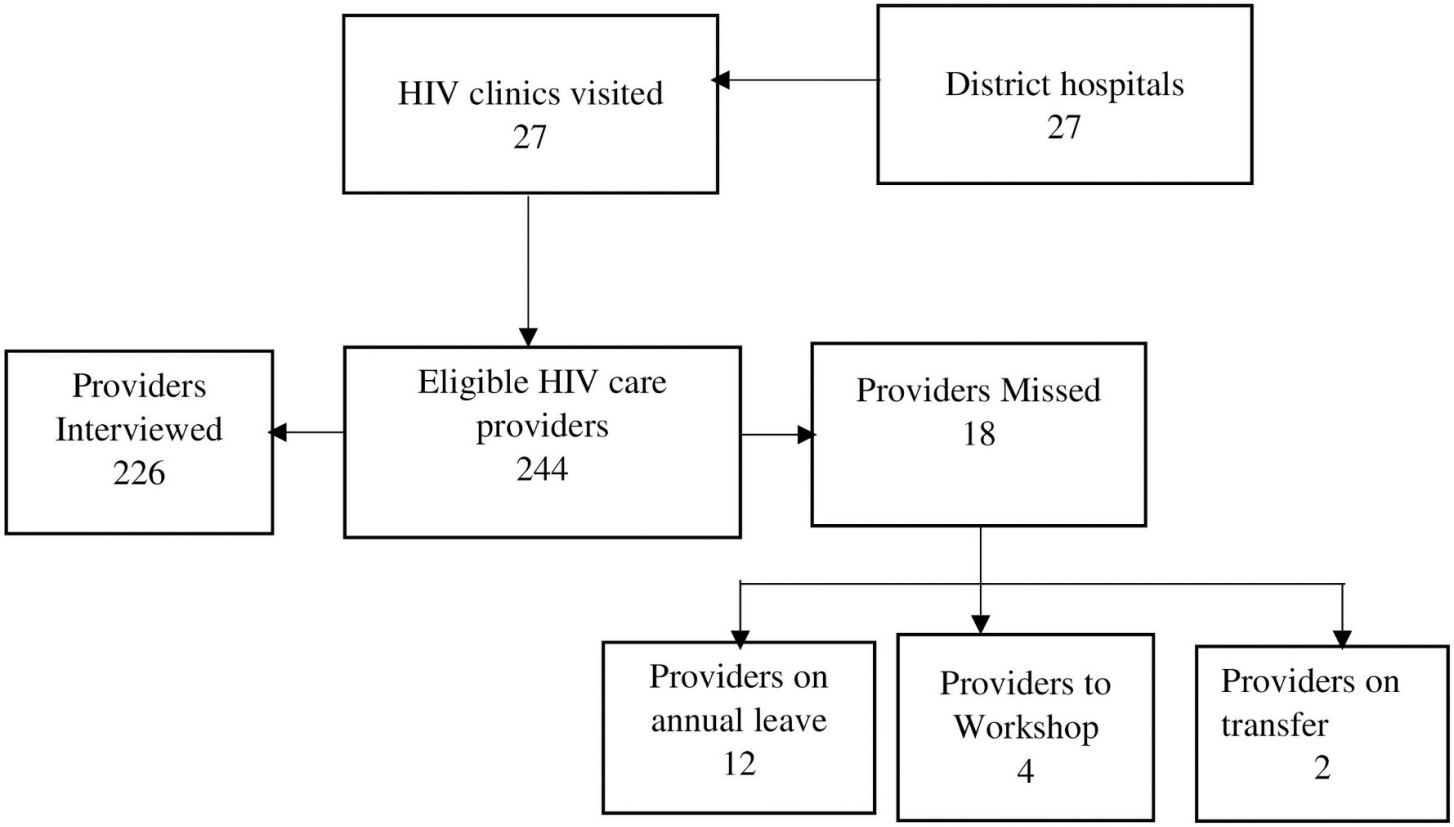
Flowchart of recruitment and study profile of the healthcare providers at the HIV clinics.

Data collection was conducted by four teams of research assistants comprising three members each. Research assistants were trained for 5 days with the fifth day used for pretesting the tools. The tools were pretested in two similar HIV clinics to ensure that measurement errors and respondent burdens are minimized and to point out problem areas for rectification. The teams collected the main data only on weekdays from 9^th^ April to 24^th^ April 2019 (12 working days). Each team visited a facility for at least five working days. Follow-ups/return visits were made for 4-days while the team was still within the facility such that every participant could be interviewed. In the end, a total of 244 healthcare providers from the 27 district hospitals across the country were established as potential participants at the HIV clinics. We interviewed 226 participants, with the number of healthcare providers interviewed per clinic ranging from 6 to 12 as shown by the flowchart of the recruitment and study profile (Fig 4).

### Data management and analysis

An EPIDATA version 3.1 software was use to capture the collected data which based on hardcopy questionnaire and further imported into Stata 15 for cleaning and analysis.

Questions covering content were measured by *1 = yes*, meaning the activity was performed or provided and *0 = no*, meaning the activity was not provided or performed. Other items under this construct were open-ended questions at the time of the data collection. They were post-coded later with a scale of 1 to 3 indicating the degree to which the provider’s response reflected adherence to what is in the intervention guideline *(1 = not adherent; 2 = partially adherent and 3 = fully adherent)*. The frequency components of the questionnaire were measured on a scale of 1 to 5 *(1 = never*, *2 = rarely*, *3 = for some clients sometimes 4 = either for all new clients or annually for existing clients*, *5 = All the time for all clients)*, with a scale 5 being what the intervention guidelines expected.

We first had to determine the level of implementation fidelity from the questions/items covering content and frequency and the further identify associated provider characteristics. The questionnaire items–which were selected from the guideline–had never been used to measure fidelity anywhere and as such, there was no reference for what would constitute high or low fidelity. There was therefore a need to identify the set of items or variables underlying the plausible factors or components required to measure provider implementation fidelity to the TB screening intervention guidelines. Since there was no existing study on how different items, number of items and components relate to one another to help us determine the fidelity level, we proceeded to arrive at a parsimonious representation of the associations among our measured variables. In order to do so and find the underlying variables for measuring fidelity to the guidelines on TB screening of PLHIV at HIV clinics, the exploratory factor analysis (EFA) method using polychoric correlations matrix technique was initially employed. On applying the EFA, we found that the factor loadings and unique variances were very low as expected but three of the 19 items. The factor analysis was however not value-adding as there was no apparent significant variable reduction. Therefore, the original items were instead used for further analysis.

As a result, we re-grouped the original items selected from the TB screening guideline into four main components based on our knowledge and experience. Each of the four components has different items that must be implemented to achieve the purpose of the TB screening intervention among PLHIV. These components are as follows:

1. *TB diagnosis* through screening for signs and symptoms of TB among all PLHV at all HIV clinics during every visit.
2. *TB Awareness* through health education on TB and counselling of all HIV clients at every HIV clinic visit.
3. *TB symptoms questionnaire* to screen all HIV clients for TB at all HIV clinics.
4. *TB screening process* which entails providers’ knowledge of the processes of early diagnosis and treatment of HIV-associated TB at the HIV clinic.

Each of the four components of the intervention is made up of between two to ten items as outlined in the guideline for implementation of TB/HIV collaborative activities [51]. Among the 19 items identified items, the TB diagnosis component comprises 10; TB awareness 4; TB symptoms questionnaire 2; and TB screening process 3 items.

To proceed with assessing the provider fidelity level, we conducted an internal consistency and reliability check to ensure that the various items are suitable to measure each of the four components. The Cronbach Alpha which measures the internal consistency (the extent to which the items in a test measure the same component) of a scale [52] was used. A calculated alpha closer to one showed items measure the component well. The calculated alpha was high (approximately 0.900) for TB diagnosis, TB awareness, and TB symptoms questionnaire components except the TB screening process with an alpha score of 0.423. The TB screening process component was therefore excluded from the analysis based on it low alpha value [52]. The Cronbach Alpha calculated for all the items from the three components (TB diagnosis, TB awareness, and TB symptoms questionnaire) showed that the data seem to fit well (Alpha = 0.915) in the scale in all aspects to enhance the accuracy of overall provider fidelity level assessment.

We carried out the analysis on the three components and their respective items. Based on the various scoring for each item, the total maximum possible score was 48 (16 items) per provider (TB diagnosis 30; TB awareness 12; and TB symptoms questionnaire 6). Item scores for each component were summed to determine component-specific fidelity scores for each HIV care provider. An overall provider implementation fidelity level was then determined by summing items scores of all the three components.

The scores of the three components and the overall were converted to proportions based on the respective total maximum possible score and summarized using Median, Inter-quartile ranges (IQR), and percentages. These scores were measuring extent of fidelity and compared across geographic location of the health facility and provider characteristics using the Kruskal Wallis and Chi square tests.

Reference point regarding what constitutes a high level of provider implementation fidelity to TB screening among PLHIV is unknown. To aid interpretation of the fidelity scores, we arbitrarily used the median fidelity score computed from this study as a cut-off, since our data was not normally distributed. Therefore we grouped as low fidelity those providers whose fidelity scores were below the median and as high fidelity those whose fidelity scores were equal or above the median fidelity (coded as *0 = low provider fidelity level* and *1 = high provider fidelity level*).

A descriptive analysis on the participants’ characteristics and levels of fidelity was conducted. Logistic regression (LR) was used to examine for association between the IF level and characteristics of the healthcare provider adjusting for the district hospital (random) effect. Statistical significance was considered at 5% level. The variance inflation factor (VIF) calculated to measure the amount of multicollinearity in the regression variables ranged between 1.02 and 1.34 (mean VIF = 1.18), which indicates no correlation between the variables. All the analysis were performed using the Stata 15 software [53].

## Results

### Descriptive analysis

#### Characteristics of healthcare providers

The study profile (Fig 4) showed that 226 HIV care providers participated in this study. The result from descriptive analysis (Table 2) found that out of the 226 healthcare providers interviewed, about 60% (135) were females. The mean age of the providers was 34.5 years (Standard Deviation [SD] = 8.3) and 65% (147) providers have worked in the HIV clinic for less than 5 years. The results also showed that 63% (142) of the providers were clinicians whilst 62% (141) had attained a post-secondary non-tertiary level of education.

**Table 2:** Characteristics of the healthcare providers by profession at the HIV clinics in Ghana, 2019.

Characteristics of providers	Clinicians	Non-clinicians	All providers
	n (%)	n (%)	n (%)
**Age in completed years, Mean (SD)**	34.6 (9.1)	34.3 (7.0)	34.5 (8.3)
**Gender**			
Female	109 (76.8)	26 (30.9)	135 (59.7)
Male	33 (23.2)	58 (69.1)	91 (40.30)
**Years working in HIV care clinic**			
<5years	102 (71.8)	45 (53.6)	147 (65.0)
5years plus	40 (28.2)	39 (46.4)	79 (35.0)
**Highest educational level**			
Upper secondary	5 (3.5)	5 (5.9)	10 (4.4)
Post-secondary non-tertiary	108 (76.1)	33 (39.3)	141 (62.4)
Tertiary	29 (20.4)	46 (54.8)	75 (33.2)
**Total**	**142 (100)**	**84 (100)**	**226 (100)**

Among the clinicians, the mean age was 34.6 years (SD = 9.1), 76.8% (109) were females and most (76.1%, n = 108) had attained post-secondary non-tertiary education level followed by tertiary education (20.4%, n = 29). Also, among the non-clinicians, the mean age was 34.3 years (SD = 7.0), most (69.1%n = 58) were males, and most (54.8%, n = 46) attained tertiary level of education followed by post-secondary no tertiary education (39.3%, n = 33).

#### Descriptive statistics of the raw items/variables

Table 3 presents the descriptive statistics for the raw data for determining implementation fidelity for the TB screening intervention. For example 73% (165) of the providers use the TB screening questionnaire at the HIV clinic during TB screening while 66% do ask all the HIV clients all the TB screening question during screening.

**Table 3:** Descriptive statistics of the fidelity assessment items among HIV healthcare providers at the HIV clinics in Ghana.

**Items/Variables**	**N = 226, n (%)**	
	**No**	**Yes**
1. Asks client about cough of any duration.	31 (13.7)	195 (86.3)
2. Asks if the client has night sweats.	71 (31.4)	155 (68.6)
3. Asks if the client had fever.	86 (38.1)	140 (61.9)
4. Asks if the client has pain in the chest.	57 (25.2)	169 (74.8)
5. Asks/checks if the client lost weight.	74 (32.7)	152 (67.3)
**6.** Provides education.	77 (34.1)	149 (56.9)
7. Provides counselling about TB screening.	62 (27.4)	164 (72.6)
8. Uses TB screening questionnaire (clinical algorithm).	61 (27.0)	165 (73.0)

	**Never**	**Rarely**	**For some clients sometimes**	**Either for all new clients or annually for existing clients**	**All the time for all clients**
9. How frequently do ask PLHIV about cough of any duration during consultation?	31 (13.7)	4 (1.8)	11 (4.9)	10 (4.4)	170 (75.2)
10. How frequently do you ask PLHIV about night sweats during consultation?	71 (31.4)	6 (2.7)	7 (3.1)	5 (2.2)	137 (60.6)
11. How frequently do you ask PLHIV if they had a fever during consultation?	86 (38.1)	5 (2.2)	7 (3.1)	3 (1.3)	125 (55.3)
12. How frequently do you ask PLHIV about pain in the chest during consultation?	57 (25.2)	7 (3.1)	6 (2.7)	5 (2.2)	151 (66.8)
13. How frequently do you ask/check the weight of PLHIV during consultation?	74 (32.7)	6 (2.7)	4(1.8)	5 (2.2)	137 (60.6)
14. How frequently do you provide education for PLHIV?	77 (34.1)	8 (3.5)	15 6.6)	3 (1.3)	123 (54.4)
15. How frequently do you provide counselling about TB screening for PLHIV?	62 (27.4)	5 (2.2)	13 (5.7)	6 (2.7)	140 (62.0)
16. How frequently do you use the TB screening questionnaire for PLHIV?	61 (27.0)	2 (0.9)	22 (9.7)	7 (3.1)	134 (59.3)

Summary statistics note: The total number of interviews with healthcare providers is 226.

### Provider implementation fidelity level

The study showed that, providers in 12 out of the 27 HIV clinics scored above the median implementation fidelity score. The results in Table 4 presents the summary statistics of provider fidelity scores for the three components of the intervention and the overall. The study showed that, the TB symptoms questionnaire component had the highest median score of 100% followed by TB diagnosis and TB awareness each scoring 83%.

**Table 4:** Summary statistics of the provider fidelity scores by the components.

Component of the TB screening intervention	Fidelity score Median (IQR)	High fidelity level	Low fidelity level
		n (%)	n (%)
**1**. TB Diagnosis	83.33 (50, 100)	126 (55.8)	100 (44.2)
**2**. TB Awareness	83.33 (50, 100)	120 (53.1)	100 (46.9)
**3**. TB symptoms questionnaire	100 (16.67, 100)	134 (59.3)	92 (40.7)
**Over all**	79.17 (58.33, 100)	119 (52.7)	107 (47.3)

The overall provider implementation fidelity score ranged from 16% to 100%. The median overall provider implementation score was 79% (IQR: 58.3, 100.0). Out of the 226 HIV care providers, 53% (n = 119) had scores above the median fidelity score.

The fidelity scores and levels for the three components were also compared.

*TB diagnosis*. The fidelity score for the component of TB diagnosis ranged from 17% to 100% with a median score of 83% (IQR: 50, 100). The study showed that about 56% (n = 126) of the HIV care providers had an implementation fidelity score above the TB diagnosis median score (high fidelity level). Providers in 16 clinics had a TB diagnosis implementation fidelity score above the median.

*TB awareness*. The result showed that, the fidelity score for the component of awareness also ranged from 17% to 100%. Its median fidelity score was also 83% (IQR: 50, 100) with about 53% (n = 120) of the health care providers scoring implementation fidelity above the median score of awareness component (high fidelity level). Provider in 13 clinics were found to have an awareness component fidelity score above the median.

*TB symptoms questionnaire*. The level of provider fidelity to implementing the TB screening questionnaire among PLHIV ranged from 17% to 100%, its median score was 100% (IQR: 17%, 100%), and most HIV care providers (59%, n = 134) were in high fidelity level category. Similarly, providers in 16 clinics were found to have an implementation fidelity score above the median for the TB diagnosis component.

#### Comparison of geographical and provider characteristics between fidelity levels

From the univariate analysis presented in Table 5, most healthcare providers in Brong Ahafo (70%), Western (88%), Greater Accra (60%), Northern (60%), and Volta (87%) regions were not adherent (low IF level) to the intervention guidelines compared to most providers in Ashanti (59%), Central (77%), Upper East (70%), Upper West (77%) and Eastern (55%) regions who implemented the intervention with high fidelity. Providers in Brong Ahafo, Western and Volta regions who were not adherent to the intervention guidelines differ significantly compared to their counterparts who were adherent to the intervention guidelines in same regions. Re-grouped regions into zones as a result of relatively small numbers did not showed statistically significant association between zonal locations and IF level–although providers in Savannah zone were about 1.6 times more likely to implement the intervention with high IF that those in the Forest zone.

**Table 5:** Comparison of geographical and provider characteristics between fidelity levels.

Characteristics of providers	Low fidelity level	High fidelity level	Row Total	crude OR	95%CI
	N (%), M (SD)	N (%), M (SD)			
**Region**					
Ashanti (Ref)	13 (40.6)	19 (59.4)	32	1.00	-
Brong Ahafo	14 (70.0)	6 (30.0)	20	0.29*	0.09–0.96
Central	7 (22.6)	24 (77.4)	31	2.35	0.78–7.04
Eastern	19 (45.2)	23 (54.8)	42	0.83	0.33–2.10
Greater Accra	12 (60.0)	8 (40.0)	20	0.46	0.14–1.43
Northern	9 (60.0)	6 (40.0)	15	0.46	0.13–1.59
Upper East	6 (30.0)	14 (70.0)	20	1.60	0.49–5.24
Upper West	3 (23.1)	10 (76.9)	13	2.28	0.52–9.92
Volta	7 (87.5)	1 (12.5)	8	0.10*	0.01–0.89
Western	17 (68.0)	8 (32.0)	25	0.32*	0.11–0.96
**Ecological zone**					
**Forest***(Brong Ahafo*, *Ashanti & Eastern)* (Ref)	46 (48.9)	48 (51.1)	94	1.00	-
**Savannah***(Upper East*, *Upper West & Northern*)	18 (37.5)	30 (62.5)	48	1.60	0.78–3.25
**Coastal***(Gt*. *Accra*, *Western*, *Central & Volta)*	43 (51.2)	41 (48.8)	84	0.91	0.51–1.65
**Mean age of provider in completed years**	33.8 (6.9)	35.1 (9.4)	-	1.02	0.98–1.05
**Years working in HIV clinic**					
<5years (Ref)	72 (49.0)	75 (51.0)	147	1.00	-
5years plus	35 (44.3)	44 (55.7)	79	1.20	0.70–2.09
**Gender**					
Male (Ref)	55 (60.4)	36 (39.6)	91	1.00	-
Female	52 (38.5)	83 (61.5)	135	2.44*	1.41–4.20
**Profession**					
Non-clinician (Ref)	50 (59.5)	34 (40.5)	84	1.00	-
Clinician	57 (40.1)	85 (59.9)	142	2.19*	1.27–3.80
**Highest educational level**					
Upper secondary (Ref)	7 (70.0)	3 (30.0)	10	1.00	-
Post-secondary non-tertiary	65 (46.1)	76 (53.9)	141	2.73*	1.08–10.98
Tertiary	35 (46.7)	40 (53.3)	75	2.67	0.64–11.10

Note: The totals are in row and therefore correspond to total percentages of 100%.

*Significant at 95%CI.

Provider IF levels significantly (p<0.05) varied in terms of gender, profession, level of education attained (tertiary level) of the healthcare provider. The results show 61% of female providers had a high level of adherence to the TB screening intervention guidelines, as did 60% of clinician providers, 40% of non-clinician providers, 54% of healthcare providers who attained post-secondary non-tertiary education, and 53% of providers with tertiary education. Female providers were about 2.4 times more likely to adhere to implementation guidelines than male counterparts (p<0.05). Also, providers who attained post-secondary non-tertiary were about 3 times more likely to implement the intervention with high IF than those who attained upper secondary (p<0.05). The study found no significant difference in provider IF levels across educational age and the number of years working at the HIV clinic (p>0.05).

### Factors associated with provider implementation fidelity level

We used the ecological zones in the multiple logistic regression instead of regions due to the small sample sizes and their potential effects on the confidence intervals. The cluster-based logistics regression result after adjusting for the district hospital as the cluster effect showed that gender, profession, and tertiary educational level of the HIV care provider were significantly associated with provider IF level (Table 6). After adjusting for other factors and the hospital effect, female providers were about 2.4 times (AOR = 2.36, 95% CI: 1.09–5.10, p = 0.029) more likely than their male counterparts to implement the TB screening intervention among PLHIV with high IF level.

**Table 6:** Multivariable logistic regression between implementation fidelity level and background factors with district hospital (random) effect.

Characteristics of providers	AOR	95% CI	p-value
**Ecological zonal**			
**Forest***(Brong Ahafo*, *Ashanti & Eastern)* (Ref)	1.00	-	-
**Savannah***(Upper East*, *Upper West & Northern*)	1.83	0.70–4.80	0.220
**Coastal***(Gt*. *Accra*, *Western*, *Central & Volta)*	0.95	0.28–3.21	0.942
**Gender**			
Male (Ref)	1.00	-	
Female	2.36	1.09–5.10	0.029**
**Profession**			
Non-clinician (Ref)	1.00	-	-
Clinician	1.78	1.07–3.50	0.045*
**Highest educational level**			
Upper secondary (Ref)	1.00	-	-
Post-secondary non-tertiary	2.66	0.48–14.66	0.260
Tertiary	4.31	2.12–9.10	0.040**

AOR = adjusted odds ratio,

* marginal significant and

** significant at 95%CI.

The odds of being a clinician (AOR = 1.78, 95%CI: 1.07–3.50, p = 0.045), and attaining tertiary education level (AOR = 4.31, 95%CI: 2.12–9.10, p = 0.040) and implementing the intervention with high compliance is higher than being a non-clinician, and attaining upper secondary educational level after adjusting for other factors and hospital effect.

## Discussion

This study aimed to assess provider IF to the guideline on TB screening among PLHIV attending HIV clinics in Ghana and potential factors associated with IF level. Despite the growing number of studies assessing fidelity of implementation in health, our study is the first in Ghana and one of the few in Africa to assess implementation outcome such as fidelity to the guideline on TB screening at the HIV clinics. Most studies conducted in this area focused on intervention outcomes such as cured, died, completed treatment, among others [42, 54]. The determination of IF scores were done the summation approach based on providers’ responses to questions on core components of the intervention [55]. The IF level refers to those providers with composite scores equal or above the median score (high IF level) vs. those with composite scores below the median score (low IF level). This approach advances methods for measuring IF by statistically defining what constitutes high versus low level of fidelity. The creation of a level of implementation (low versus high implementation) is one of the two ways of assessing fidelity [15, 56], that is rarely applied in the IS literature.

Based on the structured questionnaires, the first key finding of this study is that, among all healthcare providers surveyed, 53% had scores equal or above the overall median fidelity score of 79%. Since there was no existing reference point of what constitutes high implementation fidelity level, using the median as a cut-off as done in our study implies that only 53% of the providers implemented the intervention with a higher level of fidelity than the 79%. The number who implemented with high fidelity was marginally (6% points) higher than those who implemented with lower fidelity score than the median. It is difficult to interpret whether a 79% fidelity score is adequate, for the TB screening intervention, in this context. This fairly high percentage of providers with lower adherence score (<79%) might lead to spurious conclusions about the effectiveness of the intervention [17, 57, 58]. Putting this finding in context, there is evidence that coverage of the intervention has been below set targets in the past years [51]. Our findings show that, overall, 47% of providers implement the intervention with low fidelity which may contribute to this sub-optimal coverage. We observed high provider fidelity scores in only 12 out of the 27 HIV clinics. Among the three components, providers in 16 clinics adhered to the items or activities constituting the TB diagnosis and the TB symptom questionnaire components. Provider non-adherence or low IF of healthcare interventions are known in the published literature to weaken chances of attaining intervention outcomes and render efficacious interventions ineffective in real life [22].

The next key finding of our study is that contextual factors (geographical location) influence the level of IF to the intervention. Even though a moderate proportion of healthcare providers implemented TB screening among PLHIV with high fidelity level overall, there were marked statistically significant differences in the level of fidelity of implementation across **some** regions. Among the 10 regions of the country at the time of the study, most healthcare providers in five regions adhered (55–77%) to the intervention guidelines whilst in the other five regions, most did not (60–88%). The urbanity of the facility where the intervention is being implemented influences IF level [13, 59, 60]. Even though our study found some relationship between the regional locations of the district hospital and IF level, it is beyond the scope of this study to delve into urbanity of the district hospitals where this study was conducted. Although the samples sizes were small, regional location is a critical factor in our analysis because the health system of Ghana has cascading administrative management from regional through the district to hospital level and implementation of new intervention are discussed and planed using the top-bottom approach. Hospitals within the same region should share similar findings from an intervention. It will be worthwhile to investigate more fully the impact of context in the implementation of this intervention. Regions were re-grouped into three ecological zones (forest, savannah and coastal) due to insufficient sample size as mentioned. After accounting for the HIV clinic as a cluster effect, none of the ecological locations of the HIV clinic was found to statistically influence the IF level. That notwithstanding, since the TB screening intervention is guided by universal national protocols and guidelines it is expected that the adherence to intervention guidelines by providers will be uniform spatially. But our study found variability in IF spatially which is similar to the variability found between primary healthcare facilities in South Africa [30]. It is therefore important that supporting activities and resources aimed at ensuring provider adherence with TB screening should be streamlined across all regions to ensure uniformity in the delivery of the intervention.

Our study found that provider characteristics influenced IF level, similar to other studies in the literature [11, 12, 15, 56]. Factors reported in the literature to influence the level of fidelity include gender, education, cadre (profession), perceived need and benefit of the intervention, and work experience [15, 24]. Regarding factors that influence level of fidelity, we assessed the demographic aspect of the provider characteristics. Our findings from the cluster-based logistic regression analysis showed that the gender, profession, educational level of the providers were statistically significantly associated with provider IF to TB screening among PLHIV. This study found that after adjusting for the cluster effect (district hospital (HIV clinic)), female providers and clinicians adhered mostly to the implementation guideline and those with higher education also complied most to the intervention guidelines. We expected provider experience to influence IF but our study did not find such an association, though another study found that experience in terms of the number of years worked at the HIV clinic may not influence IF [60]. They argued that though providers experience is worth considering for implementation, in their study experience could not have predicted fidelity of implementation because most, if not all, healthcare providers had adequate levels of education and experience [60].

### Strengths and limitation

To our knowledge, this study is one of the first that assess provider implementation fidelity to the TB screening intervention in sub-Sahara Africa particularly in the context of Ghana. This study thus assessed implementation outcome, whereas most studies assess the effectiveness of the intervention in terms of the intervention outcomes. This study was a census-based survey of all HIV care providers at all the study sites which allowed us to make a good conclusion. In an attempt to reduce and combine data, we attempted a robust analytical approach through a factor analytic technique to determine factors underlying assessment of IF of the intervention. This approach did not add any value to the analysis. We therefore resorted to using original items to assess fidelity. Finally, modified CFIF directed the assessment of contextual and demographics factors found to relate to IF. The VIF showed that multicollinearity was not an issue of concerned in the logistic model.

The study also had some limitations. It assessed implementation fidelity using provider self-reported responses on how TB screening intervention among PLHIV is being implemented. Like all other studies of this nature, there is a major limitation with regards to the validity and accuracy of self-reported responses due to possible social desirability and recall bias [56, 61–63]. However, since providers interviewed were all active and currently delivering the intervention, issues with recall errors were highly minimized. Also, this method offers a time and cost-efficient means to obtain clinical and program level information from the provider perspective compared to methods that involve independent observations [22]. We had a challenge with the categorization of fidelity levels because there are no existing norms or references. We, therefore, chose the median as the cut-off because our data was not normally distributed. Median as a cut-off might lead to loss of information and reducing the statistical power to detect a relation between the variables and fidelity level, hence some of the findings should be interpreted with caution. Notwithstanding, the median serves as a yardstick and lesson learned can inform better implementation of the intervention in this and other settings. Also, categorizing fidelity into high or low simplified the statistical analysis which led to easy interpretation and presentation of our result.

## Conclusion

IF studies are important yet there are no existing norms for its categorization. This study added to knowledge another way of determining IF levels and information needed to meaningfully conclude on the performance of the TB screening intervention implemented over a decade in Ghana.

The study suggests that a high level of IF to the intervention was marginally achieved, i.e. a little above the median fidelity score for each of the three components as well as the overall. We, therefore, found elements of healthcare provider adherence as well as some evidence of low IF among healthcare providers delivering TB screening intervention to PLHIV. Some researchers have referred to these bottlenecks in implementation fidelity as type III errors [58, 64] and argue that such errors can negatively affect intervention effectiveness. Nevertheless, regional location, gender, profession, and level of education were associated significantly with IF levels in this study. Supporting strategies such as continuous training, coaching and program review among providers and program managers should be at the core of program implementation in this context. Strategies for monitoring and measuring implementation fidelity should be clearly defined and implemented to offer constant program performance information to serve as the basis for recommendations to improve program implementation outcomes.

In summary, our study demonstrated a systematic way of assessing fidelity of implementation at the provider level using the core components of the intervention. IF assessment should be encouraged or incorporated into government or national interventions to understand the weakness and areas of the intervention that needs improvement to be able to keep to desire outcomes of interventions. Most fidelity assessments were done as such but implementation scientists should consider assessing fidelity at the health facility level. This is where resources and guidelines are discussed and discharged to providers. Finally, since the providers did not fully adhered to three components of the intervention, it however indicated an opportunity for improvement of the current implementation of the TB screening intervention among PLHIV attending HIV clinics.

## Supporting information

S1 File Supporting dataset. (ZIP)

## References

[1] Date A, Modi S. TB Screening Among People Living With HIV/AIDS in Resource-Limited Settings. JAIDS J Acquir Immune Defic Syndr. 2015 Apr 15;68:S270–3. doi: 10.1097/QAI.0000000000000485 25768866

[2] Getahun H, Van Gorkom J, Harries A, Harrington M, Nunn P, Perriens J, et al. INTERIM POLICY ON COLLABORATIVE TB/HIV ACTIVITIES. TB/HIV Research FoundationRegional Office for Africa South Africa). Paul Nunn Paul Pronyk; 2004.

[3] WHO. WHO policy on collaborative TB/HIV activities Guidelines for national programmes and other stakeholders. 2012. 23586124

[4] Gupta S, Granich R, Date A, Lepere P, Hersh B, Gouws E, et al. Review of policy and status of implementation of collaborative HIV-TB activities in 23 high-burden countries. Int J Tuberc Lung Dis. 2014 Oct 1;18(10):1149–58. doi: 10.5588/ijtld.13.0889 25216827

[5] Getahun H, Gunneberg C, Granich R, Nunn P. HIV Infection–Associated Tuberculosis: The Epidemiology and the Response. Clin Infect Dis. 2010 May 15;50(s3):S201–7. doi: 10.1086/651492 20397949

[6] Ghana Health Service. Implementation of TB / HIV collaborative activities in Ghana: Technical policy and guidelines. 2007.

[7] Ghana Health Service. Implementation of TB/HIV collaborative activities in Ghana: Joint programme planning policy and guidelines. 2014.

[8] Abebe R, Fantahun M, Hailemicheal Y, Yizengaw M, Zegeye MM. Hiv testing and counseling among patients with tuberculosis at arbaminch hospital, Southern Ethiopia. Am J Trop Med Hyg. 2013;89(5):232.23817332

[9] World Health Organisation. Tuberculosis country profiles (Ghana). 2020.

[10] World Health Organization W. GLOBAL TUBERCULOSIS REPORT 2020. 2020.

[11] Hasson H. Systematic evaluation of implementation fidelity of complex interventions in health and social care. Implement Sci. 2010 Sep 3;5(1):67. doi: 10.1186/1748-5908-5-67 20815872 PMC2942793

[12] Hasson H, Blomberg S, Dunér A. Fidelity and moderating factors in complex interventions: a case study of a continuum of care program for frail elderly people in health and social care. Implement Sci. 2012 Mar 22;7(1):23. doi: 10.1186/1748-5908-7-23 22436121 PMC3342887

[13] Nurjono M, Shrestha P, Ang IYH, Shiraz F, Yoong JSY, Toh SAES, et al. Implementation fidelity of a strategy to integrate service delivery: learnings from a transitional care program for individuals with complex needs in Singapore. BMC Health Serv Res. 2019 Mar 19;19(1):177. doi: 10.1186/s12913-019-3980-x 30890134 PMC6425607

[14] Nilsen P, Bernhardsson S. Context matters in implementation science: A scoping review of determinant frameworks that describe contextual determinants for implementation outcomes. Vol. 19, BMC Health Services Research. BioMed Central Ltd.; 2019. p. 189. doi: 10.1186/s12913-019-4015-3 30909897 PMC6432749

[15] Durlak JA, DuPre EP. Implementation matters: A review of research on the influence of implementation on program outcomes and the factors affecting implementation. Am J Community Psychol. 2008 Jun;41(3–4):327–50. doi: 10.1007/s10464-008-9165-0 18322790

[16] Eboreime EA, Eyles J, Nxumalo N, Eboreime OL, Ramaswamy R. Implementation process and quality of a primary health care system improvement initiative in a decentralized context: A retrospective appraisal using the quality implementation framework. Int J Health Plann Manage. 2019 Jan 1;34(1):e369–86. doi: 10.1002/hpm.2655 30216529

[17] Department of Health and Human Services. USA. THE IMPORTANCE OF QUALITY IMPLEMENTATION FOR RESEARCH. 2013;(February).

[18] Durlak JA. The importance of implementation for research, practice, and policy. Child Trends. 2011;34:1–10.

[19] Dusenbury L, Brannigan R, Hansen WB, Walsh J, Falco M, Ghana NACP, et al. Quality of implementation: developing measures crucial to understanding the diffusion of preventive interventions. Who. 2014 Jun 1;15(1):1–9. 10.1093/her/cyg134 15522898

[20] Roberts G (Gregory J.), Vaughn S, Beretvas SN, Wong V. Treatment fidelity in studies of educational intervention. Routledge; 2017.

[21] Mihalic S. THE IMPORTANCE OF IMPLEMENTATION FIDELITY. 2002.

[22] Breitenstein SM, Gross D, Garvey CA, Hill C, Fogg L, Resnick B. Implementation fidelity in community-based interventions. Res Nurs Health. 2010 Apr;33(2):164–73. doi: 10.1002/nur.20373 20198637 PMC3409469

[23] Mowbray CT, Holter MC, Teague GB, Bybee D. Fidelity criteria: Development, measurement, and validation. Am J Eval. 2003 Sep 30;24(3):315–40.

[24] Carroll C, Patterson M, Wood S, Booth A, Rick J, Balain S. A conceptual framework for implementation fidelity. Implement Sci. 2007 Dec;2(1):1–9. doi: 10.1186/1748-5908-2-40 18053122 PMC2213686

[25] Flottorp SA, Oxman AD, Krause J, Musila NR, Wensing M, Godycki-Cwirko M, et al. A checklist for identifying determinants of practice: A systematic review and synthesis of frameworks and taxonomies of factors that prevent or enable improvements in healthcare professional practice. Vol. 8, Implementation Science. 2013. doi: 10.1186/1748-5908-8-35 23522377 PMC3617095

[26] VanDevanter N, Kumar P, Nguyen N, Nguyen L, Nguyen T, Stillman F, et al. Application of the Consolidated Framework for Implementation Research to assess factors that may influence implementation of tobacco use treatment guidelines in the Viet Nam public health care delivery system. Implement Sci. 2017 Dec 28;12(1):27. doi: 10.1186/s13012-017-0558-z 28241770 PMC5330005

[27] Mihalic Sharon F.; Muller J. Blueprints, a Violence Prevention Initiative—Google Books.

[28] Hulleman Chris S; Rimm-Kaufman Sara E; Abry T. Innovative methodologies to explore implementation: Whole-part-whole—Construct validity, measurement, and analytical issues for intervention fidelity. In Halle T., Metz A., & Martinez-Beck I. (Eds.), Applying implementation science in early childhood prog. APA. 2013;

[29] Brown TA. Confirmatory Factor Analysis for Applied Research.

[30] Lebina L, Alaba O, Ringane A, Hlongwane K, Pule P, Oni T, et al. Process evaluation of implementation fidelity of the integrated chronic disease management model in two districts, South Africa. BMC Health Serv Res. 2019 Dec 16;19(1):1–14. doi: 10.1186/s12913-018-3827-x 31842881 PMC6916104

[31] Cochrane LJ, Olson CA, Murray S, Dupuis M, Tooman T, Hayes S. Gaps between knowing and doing: Understanding and assessing the barriers to optimal health care. J Contin Educ Health Prof. 2007 Mar;27(2):94–102. doi: 10.1002/chp.106 17576625

[32] Lönnroth K, Corbett E, Golub J, Godfrey-Faussett P, Uplekar M, Weil D, et al. Systematic screening for active tuberculosis: Rationale, definitions and key considerations. Vol. 17, International Journal of Tuberculosis and Lung Disease. 2013. p. 289–98. doi: 10.5588/ijtld.12.0797 23407219

[33] Peters DH, Tran NT, Adam T. Implementation Research in Health: A Practical Guide. Who. 2013;69.

[34] Roberts Greg; Vaughn Sharon; Beretvas S Natasha; Wong V. (PDF) Measuring Fidelity in Educational Settings. Routledge; 2017. 1–150 p.

[35] Naleppa MJ, Cagle JG. Treatment Fidelity in Social Work Intervention Research: A Review of Published Studies.

[36] Durak U, Schmidt A, Pawletta T. Model-Based Testing for Objective Fidelity Evaluation of Engineering and Research Flight Simulators. In American Institute of Aeronautics and Astronautics (AIAA); 2015.

[37] Albert A, Hallowell MR, Kleiner B, Chen A, Golparvar-Fard M. Enhancing construction hazard recognition with high-fidelity augmented virtuality. J Constr Eng Manag. 2014 Jul 1;140(7).

[38] Kok MSY, Jones M, Solomon-Moore E, Smith JR. Implementation fidelity of a voluntary sector-led diabetes education programme. Health Educ. 2018;118(1):62–81.

[39] Schmidt B, Watt K, McDermott R, Mills J. Assessing the link between implementation fidelity and health outcomes for a trial of intensive case management by community health workers: A mixed methods study protocol. BMC Health Serv Res. 2017 Jul;17(1):490. doi: 10.1186/s12913-017-2320-2 28716135 PMC5512738

[40] Ahorlu CK, Bonsu F. Factors affecting TB case detection and treatment in the Sissala East District, Ghana. J Tuberc Res. 2013;01(03):29–36.

[41] Addo KK, Ampofo WK, Owusu R, Bonsu C, Nartey N, Mensah GI, et al. First Nationwide Survey of the Prevalence of TB/HIV Co-Infection in Ghana. J Tuberc Res. 2018 May 9;06(02):135–47.

[42] Ansa GA, Sifa JS. Tuberculosis and HIV integration in sub-Saharan Africa. Asian Pacific J Trop Dis. 2015;5(11):841–9.

[43] Ansa GA, Walley JD, Siddiqi K, Wei X. Assessing the impact of TB/HIV services integration on TB treatment outcomes and their relevance in TB/HIV monitoring in Ghana. Infect Dis Poverty. 2012;1(1):1. 23849044 10.1186/2049-9957-1-13 PMC3710204

[44] Sidani S. Fidelity of Intervention Implementation: A Review of Instruments. Health (Irvine Calif). 1980;7:1687–95.

[45] Breitenstein SM, Fogg L, Garvey C, Hill C, Resnick B, Gross D. Measuring Implementation Fidelity in a Community-Based Parenting Intervention. Nurs Res. 2010 May;59(3):158–65. doi: 10.1097/NNR.0b013e3181dbb2e2 20404777 PMC3421455

[46] Clark A, Breitenstein S, Martsolf DS, Winstanley EL. Assessing Fidelity of a Community-Based Opioid Overdose Prevention Program: Modification of the Fidelity Checklist. J Nurs Scholarsh. 2016 Jul;48(4):371–7. doi: 10.1111/jnu.12221 27376347

[47] Hernandez M, Gomez A, Lipien L, Greenbaum PE, Armstrong KH, Gonzalez P. Use of the System-of-Care Practice Review in the National Evaluation. J Emot Behav Disord. 2001 Jan 14;9(1):43–52.

[48] Elbeck M. An approach to client satisfaction measurement as an attribute of health service quality. Health Care Manage Rev. 12(3):47–52. doi: 10.1097/00004010-198701230-00009 3623908

[49] Steiber SR. Preventing pitfalls in patient surveys. Health Care Strateg Manage. 1989 May 1;7(5):13–6. 10293191

[50] Blodgett JG, Hill DJ, Tax SS. The effects of distributive, procedural, and interactional justice on postcomplaint behavior. J Retail. 1997 Jun 1;73(2):185–210.

[51] Ghana Health Service. IMPLEMENTATION OF TB/HIV COLLABORATIVE ACTIVITIES IN GHANA: JOINT PROGRAMME PLANNING POLICY AND GUIDELINES IMPLEMENTATION OF TB/HIV COLLABORATIVE ACTIVITIES IN GHANA JOINT PROGRAMME PLANNING POLICY AND GUIDELINES. Accra; 2014.

[52] Tavakol M, Dennick R. Making sense of Cronbach’s alpha. Int J Med Educ. 2011 Jun 27;2:53–5. doi: 10.5116/ijme.4dfb.8dfd 28029643 PMC4205511

[53] StataCorp. Stata Statistical Software: Release 15. College Station, TX: StataCorp LLC. 2017.

[54] Osei E, Der J, Owusu R, Kofie P, Axame WK. The burden of HIV on Tuberculosis patients in the Volta region of Ghana from 2012 to 2015: Implication for Tuberculosis control. BMC Infect Dis. 2017;17(1):1–9. 28724359 10.1186/s12879-017-2598-z PMC5517831

[55] Abry T, Hulleman CS, Rimm-Kaufman SE. Using Indices of Fidelity to Intervention Core Components to Identify Program Active Ingredients. Am J Eval. 2015 Sep 6;36(3):320–38.

[56] James Bell A. Evaluation Brief: Measuring Implementation Fidelity. James Bell Assoc. 2009;(October):1–6.

[57] Lewis CC, Fischer S, Weiner BJ, Stanick C, Kim M, Martinez RG. Outcomes for implementation science: an enhanced systematic review of instruments using evidence-based rating criteria. Implement Sci. 2015 Dec 4;10(1):155. doi: 10.1186/s13012-015-0342-x 26537706 PMC4634818

[58] Sánchez V, Steckler A, Nitirat P, Hallfors D, Cho H, Brodish P. Fidelity of implementation in a treatment effectiveness trial of Reconnecting Youth. 10.1093/her/cyl052 16807378

[59] Crosse S, Williams B, Hagen CA, Harmon M, Ristow L, DiGaetano R, et al. Prevalence and Implementation Fidelity of Research-Based Prevention Programs in Public Schools: Final Report.

[60] Klimes-Dougan Bonnie, August Gerald J., Lee Chih-Yuan Steven, Realmuto George M., Bloomquist Michael L., Horowitz Jason L., et al. Practitioner and site characteristics that relate to fidelity of implementation: The Early Risers prevention program in a going-to-scale intervention trial. Prefessional Psychol Res Pract. 2009;40(No. 5):467–75.

[61] Breitenstein SM, Gross D, Garvey CA, Hill C, Fogg L, Resnick B. Implementation fidelity in community-based interventions. Res Nurs Heal. 2010 Apr;33(2):164–73. doi: 10.1002/nur.20373 20198637 PMC3409469

[62] Damschroder LJ, Aron DC, Keith RE, Kirsh SR, Alexander JA, Lowery JC. Fostering implementation of health services research findings into practice: a consolidated framework for advancing implementation science. 2009;15:1–15. 10.1186/1748-5908-4-50 PMC2736161 19664226

[63] Ennett ST, Haws S, Ringwalt CL, Vincus AA, Hanley S, Bowling JM, et al. Evidence-based practice in school substance use prevention: fidelity of implementation under real-world conditions. Health Educ Res. 2011 Apr;26(2):361–71. doi: 10.1093/her/cyr013 21382882 PMC3061047

[64] Dobson KS, Singer AR. Definitional and Practical Issues in the Assessment of Treatment Integrity. Clin Psychol Sci Pract. 2006 May 11;12(4):384–7.

